# Protocol for a randomized controlled trial of an internet-based prevention intervention for young children at-risk for functional abdominal pain

**DOI:** 10.1186/s13063-024-08371-8

**Published:** 2024-08-19

**Authors:** Rona L. Levy, Tasha B. Murphy, Miranda A. L. van Tilburg, Margaret R. Kuklinski, Jennifer A. Bailey, Homer Aalfs, Isabel Badillo, Hafsah Diakhate, Tonya M. Palermo

**Affiliations:** 1 School of Social Work, University of Washington, Seattle, WA USA; 2 Cape Fear Valley Medical Center, Fayetteville, NC USA; 3 University of Washington, Seattle, WA USA; 4 Center for Functional GI & Motility Disorders, University of North Carolina at Chapel Hill, Chapel Hill, NC USA; 5 Social Development Research Group, University of Washington, Seattle, WA USA; 6 Center for Child Health, Behavior, & Development, Seattle Children’s Research Institute, Seattle, WA USA; 7 Department of Anesthesiology & Pain Medicine, University of Washington School of Medicine, Seattle, WA USA

**Keywords:** Irritable bowel syndrome, Pediatric, Abdominal pain, Child, Parent, Prevention intervention, Internet interventions, Psychosocial intervention, Social learning, Cognitive behavioral therapy, Risk factors, Illness behavior, Solicitous behavior, Protective factors

## Abstract

**Background:**

Chronic pain often clusters in families, where parents and their offspring both experience chronic pain conditions. Young children of parents with irritable bowel syndrome (IBS) represent an at-risk group for the development of abdominal pain, disability, and excess health care visits in later childhood. Parental solicitous responses to children’s expressions of discomfort and maternal modeling of their own illness behavior contribute to a greater focus on somatic sensations, leading to illness behaviors in children. This randomized controlled trial will test the effectiveness of an early preventive web-based psychosocial intervention (REACH)[TM] vs. an educational web-based safety comparison condition delivered to parents with IBS to alter parental responses and lead to improved child health and decreased health care costs.

**Methods:**

Parents with IBS who have children ages 4–7 years are recruited via community-based approaches (e.g., social media advertisements, school electronic distribution, research networks) and health care providers. The target sample is 460 parents randomized to REACH, a web-based social learning and cognitive behavior therapy (SLCBT) intervention or an educational web-based safety comparison condition (EC). Participants will be assessed at baseline, 6-week (immediate post-intervention), 6-month, 12-month, and 18-month follow-up periods (months post-completion of intervention). The primary outcome is change in parental solicitous/protective behaviors. Secondary outcomes include parent risk and protective factors, child health and symptom outcomes, and health care utilization and cost savings.

**Discussion:**

This study adapts a validated, parent-delivered intervention to treat chronic pain in children to a web-based application designed to prevent the development of chronic pain in very young, high-risk children. If successful, this strategy can both prevent adverse sequelae of this condition from developing as well as be widely accessible. Furthermore, the availability of a prevention model for parent training could result in significant short- and long-term health benefits across a broad spectrum of conditions.

**Trial registration:**

ClinicalTrials.gov NCT05730491. Registered on February 15, 2023.

**Supplementary Information:**

The online version contains supplementary material available at 10.1186/s13063-024-08371-8.

## Introduction

### Background and rationale {6a}

How people perceive and react to somatic sensations, both acute and chronic, has sometimes been described as “illness behavior” [1]. Inappropriate illness behavior may include over and underreacting to somatic sensations, worrying, focusing, and reacting to somatic sensations. Misinterpretation of normal somatic sensations as symptoms of disease may also result in seeking unnecessary medical treatment (for oneself or one’s child) for minor complaints. In the case of children, inappropriate parental concerns about children’s expressions of discomfort may also contribute to an apparent worsening of symptoms and increased disability [2].

Unexplained symptoms and associated illness behavior in children are prevalent, disabling, and costly. Abdominal pain, the second most common recurrent pain complaint of childhood (after headaches), affects approximately 13.5% of children worldwide [3]. Frequent pain is associated with increased school absenteeism and missed work by parents [3, 4]. The majority of children with persistent abdominal pain meet criteria for functional abdominal pain disorder (FAPD), defined as episodic or continuous abdominal pain without evidence of an inflammatory, anatomic, metabolic, or neoplastic process that could explain symptoms [5]. FAPD is associated with significant illness behavior such as disruption of activity and high health care utilization, resulting in low health-related quality of life (HRQOL), as well as emotional distress in both children and parents [4, 6–14].

Familial clustering of idiopathic chronic abdominal pain has been observed [15]. Lewis et al. found that children whose mothers have a history of IBS are 62% more likely to report symptoms of gastrointestinal disease in the absence of physiological findings than those without IBS [16]. Walker and Greene [17] found a significant positive association between severity of somatic symptoms in children diagnosed with FAPD and similar symptoms in both mothers and fathers, suggesting that either parent may influence the development or maintenance of these symptom reports. A study by Levy et al. that examined idiopathic chronic abdominal pain rates between monozygotic and dizygotic twins and their parents concluded that the contribution of learning processes appeared to be greater than that of genetics in the development of idiopathic chronic abdominal pain [18].

There is mounting evidence that multiple factors, including parental modeling, solicitous responses, and beliefs, account for the development and maintenance of symptom reports in children with a diagnosis of FAPD [19–24]. Learning theory [25, 26] provides a strongly supported explanatory model for why gastrointestinal complaints may run in families. Children may learn from their parents by observing how parents react to their own illness symptoms (modeling) and/or by how parents respond to the children’s gastrointestinal symptoms (concern and reinforcement). Several randomized controlled trials (RCTs) have demonstrated the effectiveness of social learning theory approaches including parent cognitive behavioral interventions (such as parental reinforcement of wellness behaviors) for children with established chronic pain conditions, including FAPD [27–30]. Therefore, psychosocial interventions have become part of the recommended treatment for FAPD [31].

There is evidence that children may incur a significant increase in the risk for developing abdominal pain and disability just prior to, or in the first years of school [16, 32], suggesting that strategies to prevent illness behaviors should be implemented before they develop or are well established. However, to date, preventive interventions have not been used to target the illness behavior patterns in childhood that are critical precursors leading to development of FAPD. Developing strategies to prevent and/or reduce illness behavior in children at risk for FAPD has the potential to mitigate deleterious effects on children’s academic, social, and emotional development [33]. Prevention studies following participants as far as late adolescence or young adulthood have demonstrated positive, long-term effects on a range of developmental outcomes from interventions that targeted parenting skills and other external or internal risk factors during early childhood [34–36].

A social learning approach, with its emphasis on parent education and skills training, provides a potentially powerful framework for early intervention with families whose children are at higher risk for illness behavior due to their parent’s IBS or idiopathic abdominal pain. Intervening early, before patterns have a chance to become established, could truncate the development of illness behaviors (e.g., somatic complaints and activity avoidance) that often lead to disability. Intervening early can also improve children’s health-related quality of life and reduce children’s healthcare utilization, especially attributable to FAPD [37]. Though beyond the scope of this study, more distal goals of early, preventative intervention include forestalling the development of FAPD, with its potentially lifelong negative effects on health and well-being. Furthermore, delivering the intervention via the internet through approaches our team has successfully used with other CBT pain interventions enhances the potential for scalability and dissemination [38, 39].

**Fig. 1:**
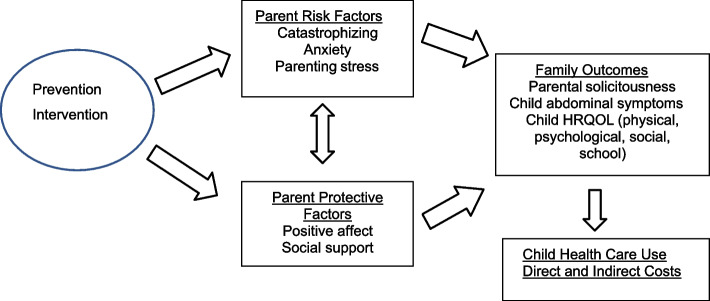
Intervention targets and outcomes

Figure 1 provides the conceptual model of the targets and outcomes of the prevention intervention on which this study is based.

Specifically, the intervention being tested here is designed to decrease risk factors for children of parents with IBS or idiopathic abdominal pain (parent catastrophic thinking about their own and their child’s symptoms and anxiety, and parenting stress) and increase protective factors (positive affect and social support). This, in turn, is expected to decrease parental solicitousness (primary outcome) as well as decrease children’s abdominal symptom complaints, and increase children’s physical, psychological, social, and school functioning, which will result in reduced health care utilization, and related direct and indirect costs including parent missed work time and costs of medical visits.

### Objectives {7}

This randomized controlled trial (RCT) aims to determine the efficacy of REACH (REsilient Active CHildren; https//reachkids.info), a web-based preventive social learning and cognitive behavior therapy (SLCBT) intervention delivered to parents with IBS, compared to an educational web-based safety comparison condition (EC) (aim 1). This trial also aims to determine the contribution of changes in parental risk and protective factors in mediating treatment effects (aim 2). Finally, we aim to determine cost savings of implementing this preventive intervention on health care expenditures and resource utilization over 18 months (aim 3).

The following hypotheses will be tested: (1) Parents receiving SLCBT will show lower solicitous behaviors (primary outcome) and lower levels of risk factors, including anxiety, parenting stress, and catastrophizing, than those receiving EC; (2) Parents receiving SLCBT will show increased levels of protective factors, including higher positive affect and social support, than those receiving EC; (3) Children of parents trained in SLCBT will have better physical, psychological, social, and school functioning, and fewer abdominal symptoms than those of parents in EC; (4) Changes in parental risk and protective factors will mediate treatment effects on child health-related quality of life; and (5) The SLCBT group will demonstrate superiority in a cost savings analysis compared to the EC group.

### Trial design {8}

**Fig. 2:**
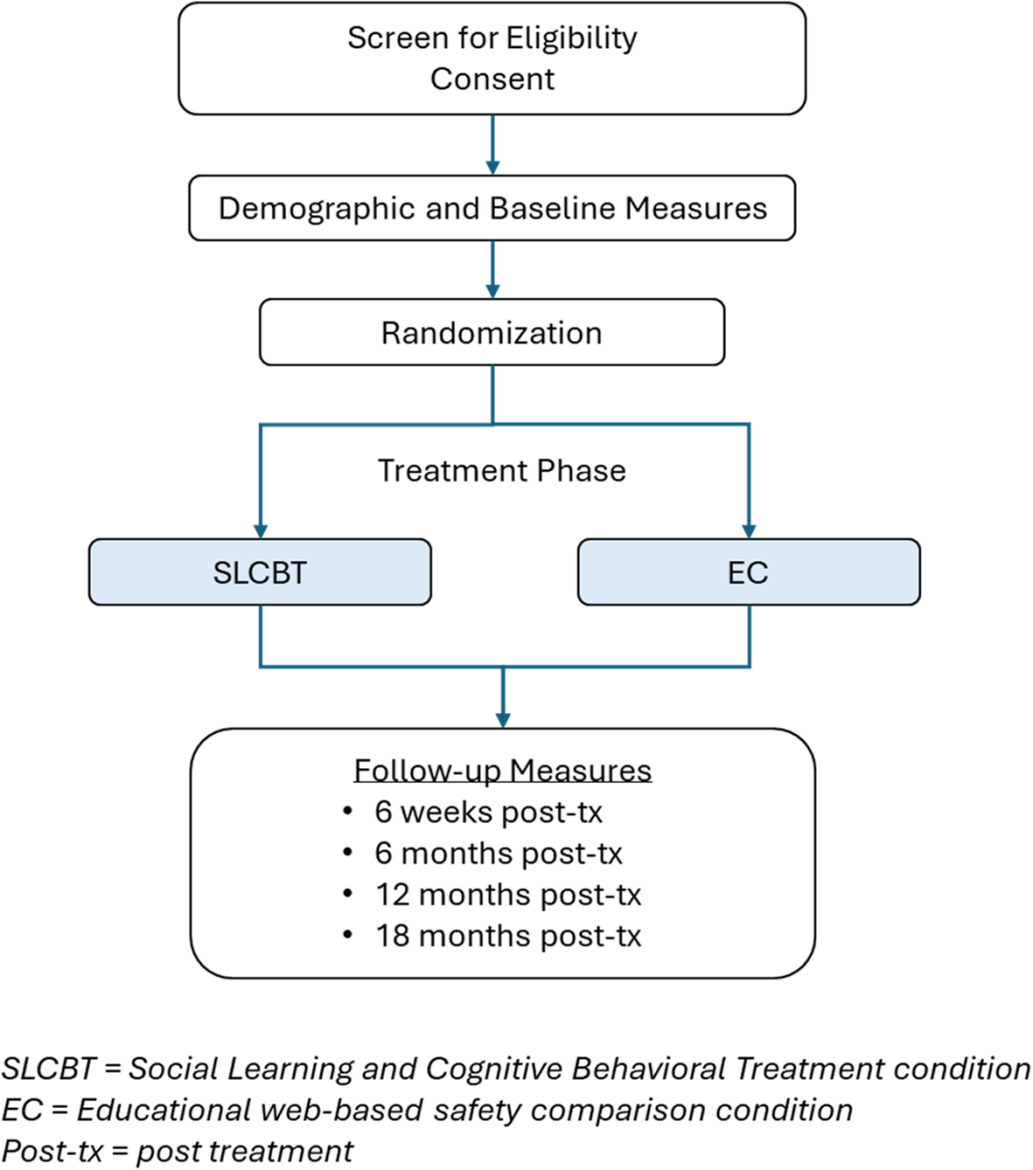
Study recruitment and measurement flowchart

This study utilizes a RCT with parallel 1:1 assignment to two study arms, (1) REACH, a SLCBT prevention intervention, and (2) an educational comparison condition (EC). Figure 2 provides the study recruitment flow and condition allocation.

## Methods: participants, interventions, and outcomes

### Study setting {9}

The study team is based at academic institutions and hospitals (University of Washington, Seattle Children’s Hospital, University of North Carolina at Chapel Hill, Cape Fear Valley Medical Center). Study participants, however, are recruited through social media outlets, newsletters, and relevant community groups (as described in Sect. 15) and may reside anywhere in the United States (because the intervention is web-based there are no limitations on participant location within the US). All participant data will be collected electronically through REDCap (Research Electronic Data Capture) [40], hosted at the University of Washington in Seattle, WA, USA.

### Eligibility criteria {10}

The study sample will include 460 parents with IBS who have young children ages 4–7 years. Complete inclusion and exclusion criteria are presented in Table 1.

**Table 1:** Eligibility Criteria

**Inclusion criteria**
•The family has at least one parent (including biological or step-parent) who a) has been diagnosed with IBS or idiopathic abdominal pain in the last five years OR meets the ROME IV criteria [41] for IBS (abdominal pain at least weekly; pain related to defecation, change in stool frequency, and change in stool form at least 30% of the time), b) is the parent primarily responsible for caring for the child on a day-to-day basis (primary caregiver), and c) is willing to participate in the study. •The child is 4 to 7 years old at the time of screening. If multiple children in this age range are present in the family, the parent will be asked to select one child for study participation. •The child must currently live at least half of the time with the parent involved in intervention, in the U.S.
**Exclusion Criteria**
•The parent is not able to read, speak, and understand English. •The child has a developmental disability that requires full-time special education, as there may be differences in these families that influence intervention response compared to typically developing populations. •The child has daily chronic pain (pain most/every day for at least 3 months). •The child has a current doctor’s diagnosis of a painful gastrointestinal disorder like functional constipation, lactose/fructose/gluten intolerance, celiac disease, Inflammatory Bowel Disorder, etc. This does not include non-painful disorders like GERD. •The child has a severe chronic disease such as juvenile arthritis, cancer, or other severe condition(s) requiring chronic medical treatment. •The parent does not have regular access to the Internet on a desktop, tablet, smartphone, or laptop computer.

### Who will take informed consent? {26a}

If families are interested and eligible to participate, study staff will complete screening and obtain consent from parents and assent from participating children ages 7 or older via online REDCap e-consent forms.

### Additional consent provisions for collection and use of participant data and biological specimens {26b}

N/A: This study does not collect biological specimens, nor is it affiliated with any ancillary studies.

### Interventions

#### Explanation for the choice of comparators {6b}

This study has two intervention groups: education control (EC) and social learning and cognitive behavioral therapy (SLCBT) (see Sect. 11a below). As this study is novel, in that preventive interventions have not previously been applied to reduce intergenerational transmission of pain conditions, there is no standard for control conditions for prevention studies of this nature. Our team, however, has developed and tested SLCBT interventions in prior studies to change parental behaviors and modeling to reduce children’s illness behaviors; the education control comparator condition in this study is similar to those we have implemented in these prior large randomized controlled trials in children with FAPD and other pain conditions. The purpose of this comparator condition is to control for time, attention, and online usage, and to allow masking to treatment allocation.

#### Intervention description {11a}

##### Education control (EC) condition

Participants assigned to the control group will receive access to a web program and complete three modules focused on child health and safety behaviors, including sports and water safety, home and fire safety, and technology safety. The EC condition is designed to be completed in a 4-week period and uses several interactive elements to increase engagement including brief questions to assess participant knowledge, question/answer components, and instructional videos. The control condition is designed to match the active intervention in time, attention, and expectancy, three major components of the placebo effect. In two previous studies, a safety focused control condition produced credible treatment expectancy effects [42, 43].

##### Social learning and cognitive behavioral therapy (SLCBT) condition

**Fig. 3:**
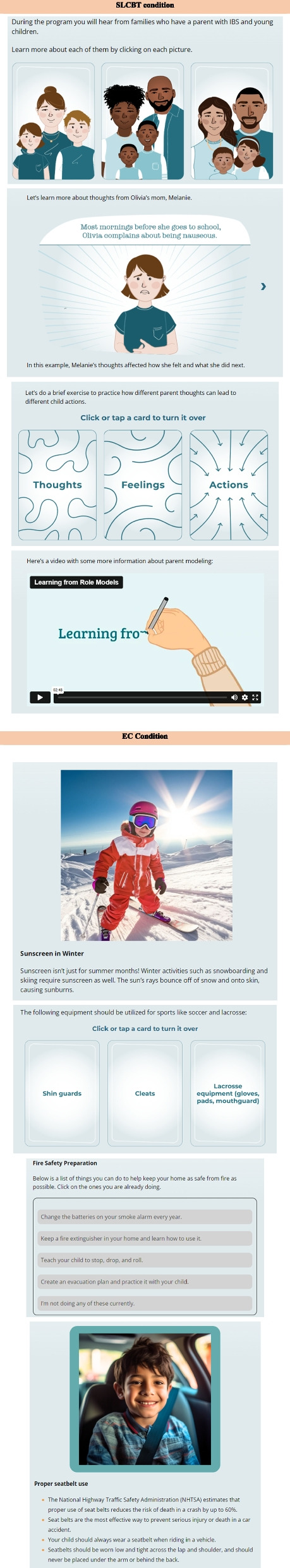
Sample screenshots of REACH intervention conditions

Participants assigned to the SLCBT group will complete a 4-week web-based intervention. The website includes an introductory module, three skills-training modules (i.e., strategies to promote wellness behaviors, use adaptive cognitive coping, model wellness behaviors for their children, and determine when it is appropriate to take further action regarding potential illness in their child), and a brief maintenance and summary module. The web modules were designed to be visually attractive and interactive in order to engage participants through using brief questions to assess participant knowledge, interactive question/answer components, instructional videos, and storyboards that participants can click through. Screenshots can be seen in Fig. 3. Additionally, participants are provided a “Toolbox” in which informational handouts can be accessed, downloaded, and printed. The handouts provide a summary of the knowledge provided in each module. Participants in each group are instructed to log in to the website for 3–4 weeks, approximately 20–30 min per week. Research team members will regularly assess web program usage for all participants and will send reminders up to two times each week as needed to encourage regular usage and completion of all modules.

### Criteria for discontinuing or modifying allocated interventions {11b}

This protocol is for a web-based prevention intervention, and as such, the intervention is not able to be modified. Participants may, however, opt out of the study at any time. There are no criteria for discontinuing intervention.

### Strategies to improve adherence to interventions {11c}

Adherence during the treatment phase is monitored as follows: Participants will receive a link to the study website via email. Participants will have the ability to log in and out of their online study dashboard as needed; when they log back into the website it will take them to the location where they left off. The study website will track participant progress and send reminder emails to complete each module if they are not completed within the module timeframe. Study staff will also track progress and send additional reminders to participants as needed to promote website usage. Once a participant has completed a module, they will receive another email informing them when their next module is ready to begin with a link to access their online study dashboard. These email contacts will ensure that accessing the study materials is convenient and efficient.

### Relevant concomitant care permitted or prohibited during the trial {11d}

Study participants are not prohibited from seeking other interventions or treatment during their participation in the study. Participants and their child(ren) can engage in any interventions (e.g., counseling, medication) as recommended by their care providers. The study team will collect information on any medical or psychosocial treatment received by the participants throughout their duration in the study.

### Provisions for post-trial care {30}

Adverse events are not expected, however, if they occur they are expected to be minor. Therefore, there are no provisions for any additional post-trial care or to provide compensation to those who suffer harm from trial participation.

### Outcomes {12}

The primary outcome is change in parental solicitous/protective behaviors. Secondary outcomes include parent risk and protective factors and child health and symptom outcomes. Participant outcomes will be assessed at baseline, immediate post-intervention (6 weeks) and at 6-month, 12-month, and 18-month follow-up periods. See also Sects. 6a and 18a.

### Participant timeline {13}

See Fig. 4.

**Fig. 4:**
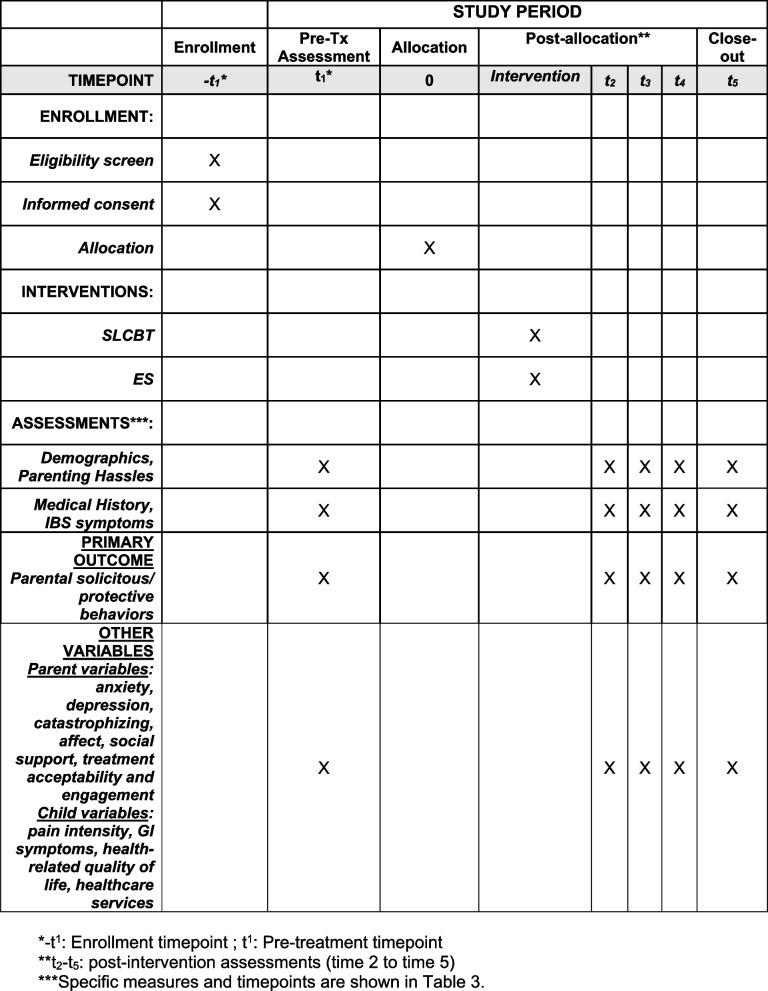
Schedule of enrollment, interventions, and assessments [13]. *-t_1_: enrollment timepoint; t_1_: pre-treatment timepoint. **t_2_–t_5_: post-intervention assessments (time 2 to time 5). ***Specific measures and timepoints are shown in Table 1

### Sample size {14}

Based on our prior studies with the intervention [43], we expect the intervention effect sizes for our primary outcome measure in the range of 0.30–0.40 (Cohen’s *d*). Using Optimal Design software [44], we calculated that a sample size of 210–380 families is sufficient to detect the expected effect sizes with a power of 0.80 and alpha = 0.05. Power to detect mediation in analyses was calculated using Monte Carlo estimation methods. For single mediator models such as those proposed here, a sample size of 368 has > 0.80 power to detect a medium sized indirect effect that explains 13% of the variance in the outcome.

### Recruitment {15}

Recruitment will be conducted through community-based approaches including health care providers, social media (e.g., postings with relevant groups and communities via Facebook, Twitter, Instagram, and others), school electronic distribution lists, and networks that connect volunteers with research projects (e.g., ResearchMatch). Potential participants complete a study interest and pre-screening form hosted on REDCap, a secure web-based data collection platform. Alternatively, they can contact study staff via a phone number or email provided in the study advertisement or through the study interest form, which provides complete details and easy links to assist in completion. A second wave of screening is conducted with interested parents wherein study staff contacts potentially eligible participants via phone call/voicemail, email, or text to see if the family is interested in hearing more about the study and assess eligibility.

### Assignment of interventions: allocation

#### Sequence generation {16a}

Group allocation will be determined using a computer-generated algorithm [45, 46] with equal assignments to each study arm, using randomly selected block sizes stratified by self-reported parent sex at birth.

#### Concealment mechanism {16b}

Allocation concealment will be ensured, as randomization does not occur until after baseline measurements have been completed.

#### Implementation {16c}

As described in Sect. 16a, the allocation sequence is generated using a computer-generated algorithm. The study coordinator(s) will screen, consent, and enroll participants, administer baseline measures (via REDCap), and initiate randomization (via REDCap).

### Assignment of interventions: blinding

#### Who will be blinded {17a}

With the exception of study coordinators and statisticians (see 17b), all other study staff will be blinded to treatment assignment. Blinding will be made possible through restricting access to participants’ treatment assignments in the study database only to staff whose role is to assign participants to interventions, contact participants throughout the intervention phase, or analyze results by treatment group. Study participants will also be blinded to treatment assignment as both treatment arms are using the same web site platform with the same branding, however participants may deduce their treatment group based on the content they receive.

#### Procedure for unblinding if needed {17b}

Study coordinators and statisticians will not be blinded to the treatment assignment. In the unlikely event that an adverse event should occur, unblinding of other study staff can be conducted by the study coordinator who assigns participants to interventions.

### Data collection and management

#### Plans for assessment and collection of outcomes {18a}

All outcome measures will be completed online independently by study participants, reducing risk of bias.

#### Measures

A list of measures with descriptions and timing of assessments can be found in Table 2.

**Table 2:** Measures by timepoint

Measure	Description	Source^a^	T1^a^	T2	T3	T4	T5
***Demographics and background measures***							
Demographics form	Demographic questionnaire to report on parent and child sex, gender, race, and ethnicity; family income; and parent education	P/C	X				
Medical history	Parent medical, psychiatric, and surgical history (including chronic conditions and chronic pain) and child medical diagnoses (including abdominal pain)	P/C	X	X	X	X	X
Irritable Bowel Syndrome Symptom Severity Scale (IBS-SSS) [47]	5-item measure which assesses parent severity and frequency of abdominal pain, bloating severity, dissatisfaction with bowel habits, and the impact of these symptoms on everyday activities	P/C	X	X	X	X	X
Daily Parenting Hassles Scale [48–50]	20-item scale that assesses parent burden in meeting the needs of their children and troublesome behavior of children. Subscales include challenging behavior and parenting tasks	P/C	X				
***Outcome and process measures***							
Adult Responses to Children’s Symptoms (ARCS), Protectiveness subscale [51, 52] (primary outcome)	15-item Protectiveness subscale assesses parental protectiveness related to child illness behavior	P/C	X	X	X	X	X
Generalized Anxiety Disorder scale (GAD-2) [53]	2-item measure used for screening of parent anxiety disorder	P/C	X	X	X	X	X
PHQ-2 [54]	2-item measure used for screening of parent depression	P/C	X	X	X	X	X
PROMIS Pain Intensity Scale—Short Form [55]	3-item parent proxy and child self-report assessing child pain intensity in past 7 days	P/C, Ch	X	X	X	X	X
Pain Catastrophizing Scale (PCS)—Short Form [56, 57]	6-item measure that assesses parent catastrophizing thoughts or feelings accompanying the experience of pain	P/C	X	X	X	X	X
Positive and Negative Affect Schedule (PANAS) [58]	One 10-item scale measures parent positive affect and the other 10-item scale measures negative affect on a 5-point Likert scale	P/C	X	X	X	X	X
Multidimensional Scale of Perceived Social Support (MSPSS) [59]	12 items that measure the extent of social support received by parent from 3 sources: friends, family, and significant others. Types of social support assessed by the MSPSS include emotional, tangible, informational, social network support, and esteem	P/C	X	X	X	X	X
Children’s Somatic Symptoms Inventory (CSSI-8) [60]	8 items assess the severity of child’s nonspecific somatic symptoms. The set of GI symptoms (pain, nausea, upset stomach) are used to measure GI symptom severity	Ch	X	X	X	X	X
ROME IV Functional Abdominal Pain Questions [61]	5 items assessing ROME IV criteria for upper and lower child functional abdominal pain—frequency, severity, location, and association with eating (lower only)	P/C	X	X	X	X	X
Pediatric Quality of Life Inventory—Short Form (PedsQL-SF) [62, 63]	Brief measure of child health-related quality of life. The 15 items in the PedsQL comprise four Generic Core Scales: Physical Functioning (5 items), Emotional Functioning (4 items), Social Functioning (3 items), and School Functioning (3 items)	P/C, Ch	X	X	X	X	X
Client Service Receipt Inventory (CSRI) [64]	Validated, comprehensive inventory of health care services incurred due to the child’s symptoms that uses a standard response timeframe of the past 6 months. Parents report on three sources of child health care costs: direct medical service use, direct non-medical costs, and indirect costs	P/C	X		X	X	X
***Treatment acceptability, satisfaction, and engagement***							
Treatment Evaluation Inventory—Short Form (TEI-SF) [65]	The TEI-SF is a 4-item measure that assesses treatment acceptability and satisfaction. 9 additional items are intended to collect feedback on the study website	P/C		X			
Treatment engagement (website)	Number of logins to the program, number of modules completed, and number of completed assignments will be measured from the admin backend of the website	N/A		X			

^a^*P/C* Parent/caregiver, *Ch *Child, *N/A *Not applicable

^a^T1*= *baseline, T2 = Post-intervention, 4-6 weeks after baseline; T3 = 6-month follow-up; T4 = 12-month follow-up,  T5 = 18-month follow-up

#### Plans to promote participant retention and complete follow-up {18b}

Participants will receive automated assessment survey invitations and reminders. Research coordinators will send reminders if assessments are not complete, until the completion of the assessment period. We will use a database checklist to ensure that all study procedures are being followed (including consents) and that all surveys are being completed according to schedule. Study staff will review study participant data and monitor parent responses. Participants will also receive gift card incentives for completing baseline and follow-up assessments. All study questionnaires will be completed online so that participants may complete them at a time and place that is convenient for them.

We will continue to collect outcome data from all participants, irrespective of intervention discontinuation or deviation from protocol. However, we will construct a variable for the amount of treatment completed (number of web-based modules) and conduct sensitivity analyses to evaluate impact on intervention outcomes.

### Data management {19}

All survey data will be collected from parents online through Research Electronic Data Capture (REDCap). Research coordinators will track assessment completion regularly, monitor for any missing data, and follow up with participants when missing data are identified. Research coordinators will also use REDCap alerts, reports, and queries to minimize missing data and ensure accuracy. Additionally, study staff will monitor for data irregularities such as skip patterns and out of range data and completion times. The REDCap database will require online sign-in with a username and password assigned to individual study staff; all data stored in REDCap will be retained on a secure server accessible only to study staff. Scoring of study measures will be done via syntax to minimize errors.

### Confidentiality {27}

The subject’s identity, research records, and personal health information will be safeguarded using secure password-protected servers. The primary source of data will come from questionnaires, which will be stored electronically in REDCap (secure password-protected database). All data will be coded with a unique participant ID. The software will be hosted on a secure, HIPAA-compliant server. Only the research team and the Seattle Children’s IRB will be able to access the participant data and information collected from this study. All research data will be de-identified at the earliest possible opportunity to promote data sharing.

Given this is an online study, it is important that user information and data be protected from theft, alteration, or unauthorized access of any kind. We have developed a User Privacy Policy and a Terms of Use Agreement for the REACH web program as per standard. Use of the web program will indicate agreement and acceptance of the Terms of Use. Only study identification numbers will be used to identify participants on the website. The web program will not collect or ask for information that can be used to identify the participant. Seattle Children’s will not sell or rent information collected by the web program to others.

### Plans for collection, laboratory evaluation, and storage of biological specimens for genetic or molecular analysis in this trial/future use {33}

N/A: As stated in Sect. 26b, no biological specimens will be collected for this study.

## Statistical methods

### Statistical methods for primary and secondary outcomes {20a}

#### General analytic procedures

All main analyses will be intention-to-treat analyses, including all participants randomized regardless of intervention compliance. Missing values due to dropouts will be imputed with well-established methods that reduce bias in estimates such as multiple imputation, full information maximum likelihood, and empirical Bayes estimation, or multiple imputations with chained equations (MICE), if a substantial amount of missing data exists.

#### Analyses by aims

Aim 1: Determine the efficacy of a preventive SLCBT intervention delivered to parents with IBS compared to a time and attention placebo/education condition (EC) at post-intervention, and at 6-, 12-, and 18-month follow-up periods. Analyses for this aim will use an intent-to-treat and dose–response approach. Intent-to-treat analyses will test associations between a dichotomous treatment assignment variable and primary (hypothesis 1) and secondary parent outcomes (hypothesis 2), as well as child health and symptom outcomes (hypothesis 3). Efficacy tests will be conducted using multilevel modeling (MLM) to maximize power and account for the longitudinal design with nesting of observations within person/family. In addition, the use of multilevel modeling allows estimation of intervention effects at a specific time point (intercept) and change over time in intervention effects (slope; time × intervention interaction). For example, to test hypothesis 1, we will estimate a series of MLMs testing the effect of the dichotomous intervention assignment variable on the level (intercept) of Adult Response to Children’s Symptoms (ARCS) scores at each time point by systematically re-centering the intercept (e.g., model 1: intercept at post treatment, model 2: intercept at 6 months, etc.). Next, we will test the association between intervention assignment and the slope of ARCS scores across time points (time × intervention interaction). Controls will include parent age and sex, as well as child sociodemographic characteristics and parent pre-treatment irritable bowel syndrome severity. Dose–response analyses also will use MLM as described above; however, the dichotomous treatment assignment variable will be replaced with a treatment engagement score (e.g., modules completed).

Aim 2: Determine the contribution of changes in parental risk and protective factors as mediating treatment effects. Hypothesis 4 states that changes in parental risk and protective factors will mediate treatment effects on child health-related quality of life. Following the procedures outlined by Muthen and colleagues [66], we will test whether and to what extent parent pain catastrophizing mediates the association between intervention group and parent solicitous responses to child symptoms. Similar models will test other mediators, as well as secondary parent and child outcomes.

Aim 3: Determine cost savings of implementing this preventive intervention on health care expenditures and resource utilization over 18 months. To assess hypothesis 5 (that the SLCBT group will demonstrate cost savings in relation to the EC group), we will first estimate total health care costs in each group, which will be the sum of direct medical costs, direct non-medical costs, and indirect costs. Direct medical costs for each group will be estimated by multiplying utilization estimates for each reported service by the service-specific unit cost from the most recent Medical Expenditure Panel Survey (MEPS) [67], and then summing the costs of all services used. Direct non-medical costs for each arm will be the sum of parent-reported out-of-pocket expenses for special foods, equipment, and transportation to medical appointments. Indirect costs for each arm will be estimated by multiplying parent-reported hours of missed work and leisure time due to caring for their child (i.e., when absent from school due to illness behavior, taking to medical appointments) by the current mean hourly wage for a US worker from the Consumer Price Index of the US Bureau of Labor Statistics [68]. Cost superiority will be indicated by the arm with lower total health care costs if arms are equal in size, or by the average health care cost per family if they are not. Should the SLCBT arm prove superior as hypothesized, intervention-related cost savings per family will be estimated as the difference in average health care costs per family (health care cost/familySLCBT − health care cost/familyEC).

### Interim analyses {21b}

N/A: As this is a low-risk behavioral intervention study, there are no plans to conduct interim efficacy or safety analyses.

### Methods for additional analyses (e.g., subgroup analyses) {20b}

All analyses are described in Sect. 20a, including subgroup analyses.

### Methods in analysis to handle protocol non-adherence and any statistical methods to handle missing data {20c}

As described in Sect. 19, study coordinators will monitor for missing data and follow up with participants when missing data are identified. As described in Sect. 20a, any missing values will be handled with well-established statistical methods, including multiple imputation, full information, or empirical Bayes estimation, or MICE.

### Plans to give access to the full protocol, participant-level data, and statistical code {31c}

The study protocol and any datasets or statistical code required to support the protocol will be supplied on request. We will also make de-identified datasets available via a public data repository (see Sect. 29).

## Oversight and monitoring

### Composition of the coordinating center and trial steering committee {5d}

N/A: This project does not include committees who are involved in trial coordination or conduct. Roles and responsibilities of study staff are outlined under “Authors’ contributions.”

### Composition of the data monitoring committee, its role and reporting structure {21a}

N/A: A data monitoring committee is not needed, as the study sponsor deemed the study as requiring only a local Safety Monitoring Committee, which we describe below in Sect. 22.

### Adverse event reporting and harms {22}

This study has been designated as minimal risk by the Seattle Children’s Institutional Review Board. Monitoring study safety will occur from the initial screening, throughout the informed consent process, and through study completion under the principal investigators’ (Palermo/Levy) supervision. A Safety Monitoring Committee (SMC) consisting of two independent investigators will review study progress and recommend appropriate action regarding adverse events or other safety issues.

In the case of an adverse event believed to be related to the study, documentation will be collected to describe the nature of the event and any associated risks. All adverse events will be discussed at weekly study meetings, and event reports will be provided to the SMC for quarterly review. The PIs will comply with all requirements of the IRB for the reporting of safety data and adverse/serious adverse events. All serious and/or unexpected problems presumed to be related to the study will be reported by the PIs to the IRB, the SMC, and the funding organization within 5 days after discovery. Dr. Levy or Palermo will retain the authority to stop or modify the trial at any time. All actions taken will be documented on a case report form.

### Frequency and plans for auditing trial conduct {23}

Data quality will be ensured by annually verifying investigator compliance with all human subjects and HIPAA requirements. Additionally, Seattle Children’s Hospital’s Office of Research Compliance performs regular audits on all research studies. Furthermore, automated rules and logic in the study tracking database will enforce compliance with IRB requirements, conformance with informed consent requirements, and adherence to study protocols.

### Plans for communicating important protocol amendments to relevant parties (e.g., trial participants, ethical committees) {25}

Any protocol modifications will be updated on ClinicalTrials.gov as needed and in the participants’ consent forms if participant activities are affected. All modifications will additionally be communicated to the study team (including co-investigators) and IRB for approval prior to implementation.

## Dissemination plans {31a}

Study results will be disseminated via publication in peer-reviewed journals, reporting on ClinicalTrials.gov within 12 months of study completion, and presentations at professional society meetings. No identifying images or other personal or clinical details of participants will be presented in reports of the trial results. A summary of the study findings will be sent via email to all study participants after the final trial endpoint completion.

## Discussion

This intervention moves a previously successful parent training program for children who were diagnosed and treated for FAPD upstream to test whether it can be used to prevent precursors to future development of FAPD among children who are at risk by virtue of their parent’s experience with IBS. It will be the first study to evaluate the potential benefit of an early preventive psychosocial intervention specifically for children at risk for a common pediatric pain condition. The online aspect of the intervention being tested is also a significant advantage and significantly increases accessibility across many population groups.

### Limitations

There are several potential limitations to consider. First is the risk of drop-out. Participants may not complete all follow-up assessments. Efforts to retain study participation over 18 months of follow-up assessments include maintaining regular contact through several forms of communication (e.g., text, email, phone), sending birthday cards to children, and reminders of upcoming assessments. Second, recruiting parents into family-based preventive programs is well known to be challenging, and rates of community-wide recruitment into universal parenting programs are typically less than 20% [69–71]. However, the pervasiveness of social media in the US and Canada [72, 73] and the ease and constancy of access via mobile devices make Facebook [74, 75] and other outreach mechanisms (e.g., Twitter, Reddit) valuable tools to increase successful recruitment of participants to research studies [76–83]. Finally, there is the potential that our findings may not generalize to some populations. Our program is only available in English, and some groups may not have access to, or be comfortable with web-based interventions of this kind. If successful, it is our expectation that other delivery formats of this program will be developed.

### Strengths

The preventive REACH intervention is based on a growing body of literature that demonstrates the effectiveness of a SLCBT intervention in helping children with established chronic pain. The intervention is delivered by web and mobile technologies, which make it easily scalable for wide dissemination. Many parents do not have access to in-person psychological services or experience other significant barriers to seeking services, particularly in low income and rural areas. Access to internet and smartphone technologies is high, even among those of low income. According to a Pew Research Center Internet Survey, 98% of adults ages 30–49 years use the internet regularly (including 91% of Black adults and 97% of Hispanic adults) [84]. Our plan to use these technologies to deliver treatment allows us to potentially also reduce disparities in care. Thus, this research reflects a practical approach to prevention. Our focus on economic costs is innovative, providing data to address the potential cost-savings of SLCBT intervention in reducing health care costs and indirect costs of parental missed work time. Our community recruitment approach provides an efficient way of reaching a broad but targeted population of parents with IBS and greatly increases external validity.

In conclusion, if successful, this study has the potential to provide a model for a preventive intervention for a wide range of pediatric health problems which can impact functioning beyond early childhood in which parental responses are influencing factors. This early intervention may avoid a lifetime trajectory of disability and its associated costs.

### Trial status

Recruitment started on October 11, 2023. The current approved protocol is version date 9/1/2023 (submission approval date: 9/27/2023; approved version at final journal submission: 9/1/2023). Recruitment is estimated to be complete by April 2027. Any protocol modifications will be updated on ClinicalTrials.gov and on the participants’ consent form if required.

### Supplementary Information

Additional file 1: SPIRIT checklist

## Data Availability

The datasets that will be generated by this study will be made available in the Eunice Kennedy Shriver National Institute of Child Health and Human Development (NICHD) data repository DASH (https://dash.nichd.nih.gov/)

## Abbreviations

SLCBT: Social learning and cognitive behavioral therapy
EC: Educational web-based safety comparison condition
FAPD: Functional abdominal pain disorder
HRQOL: Health-related quality of life
RCT: Randomized controlled trial
REACH: REsilient Active CHildren
SCH: Seattle Children’s Hospital
REDCap: Research Electronic Data Capture
IBS-SSS: Irritable Bowel Syndrome Symptom Severity Scale
ARCS: Adult Responses to Children’s Symptoms
GAD-2: Generalized Anxiety Disorder scale
PCS: Pain Catastrophizing Scale
PANAS: Positive and Negative Affect Schedule
MSPSS: Multidimensional Scale of Perceived Social Support
CSSI-8: Children’s Somatic Symptoms Inventory
PedsQL-SF: Pediatric Quality of Life Inventory—Short Form
CSRI: Client Service Receipt Inventory
TEI-SF: Treatment Evaluation Inventory—Short Form
MICE: Multiple Imputations with Changed Equations
MEPS: Medical Expenditure Panel Survey
SMC: Safety Monitoring Committee
